# What patients and caregivers want to know when consenting to the use of digital behavioral markers

**DOI:** 10.1038/s44277-024-00022-9

**Published:** 2024-12-06

**Authors:** Anika Sonig, Christine Deeney, Meghan E. Hurley, Eric A. Storch, John Herrington, Gabriel Lázaro-Muñoz, Casey J. Zampella, Birkan Tunc, Julia Parish-Morris, Jenny Blumenthal-Barby, Kristin Kostick-Quenet

**Affiliations:** 1 Center for Medical Ethics and Health Policy, Baylor College of Medicine, Houston, TX USA; 2 Menninger Department of Psychiatry and Behavioral Sciences, Baylor College of Medicine, Houston, TX USA; 3 Department of Child and Adolescent Psychiatry and Behavioral Sciences, Children’s Hospital of Philadelphia, Philadelphia, PA USA; 4 Department of Psychiatry, Perelman School of Medicine at the University of Pennsylvania, Philadelphia, PA USA; 5 Center for Bioethics, Harvard Medical School, Boston, MA USA

**Keywords:** Ethics, Translational research

## Abstract

Artificial intelligence (AI)-based computational tools for deriving digital behavioral markers are increasingly able to automatically detect clinically relevant patterns in mood and behavior through algorithmic analysis of continuously and passively collected data. The integration of these technologies into clinical care is imminent, most notably in clinical psychology and psychiatry but also other disciplines (e.g., cardiology, neurology, neurosurgery, pain management). Meanwhile, ethical guidelines for implementation are lacking, as are insights into what patients and caregivers want and need to know about these technologies to ensure acceptability and informed consent. In this work, we present qualitative findings from interviews with 40 adolescent patients and their caregivers examining ethical and practical considerations for translating these technologies into clinical care. We observed seven key domains (in order of salience) in stakeholders’ informational needs: (1) clinical utility and value; (2) evidence, explainability, evaluation and contestation; (3) accuracy and trustworthiness; (4) data security, privacy, and misuse; (5) patient consent, control, and autonomy; (6) physician-patient relationship; and (7) patient safety, well-being, and dignity. Drawing from these themes, we provide a checklist of questions, as well as suggestions and key challenges, to help researchers and practitioners respond to what stakeholders want to know when integrating these technologies into clinical care and research. Our findings inform participatory approaches to co-designing treatment roadmaps for using these AI-based tools for enhanced patient engagement, acceptability and informed consent.

## Lay Summary

This study examines what adolescent patients and their caregivers need to know when agreeing to AI-based digital tools that monitor behavior for mental health care. Interviews with 40 participants revealed key areas where patients and caregivers had questions, including data privacy, clinical benefits, and control over data. These insights help healthcare providers address patient concerns, improve understanding, and ensure informed consent as these technologies become more common in clinical settings.

## Introduction

Artificial-intelligence (AI) based computer perception (CP) technologies, including digital phenotyping [1–4], affective computing [5, 6], and computational behavioral analysis [7–9], are increasingly integrated into clinical decision-making across clinical domains, most notably in psychology and psychiatry [10]. These technologies use passive, continuous data collection via device sensors (e.g., microphones or accelerometers on smartphones and wearables) to provide insights into patients’ emotional and behavioral functioning during clinical assessments, research, and daily life. Depending on the context, clinical condition, or type of devices employed, a wide range of personal data can be collected, including those not directly related to the main study/clinical assessment, such as visual appearance, movements and morphology, speech, geolocation, accelerometry, phone screen capture/usage, call and text logs, social media content, physiological and even neural activity [6]. This is especially true for devices employed outside of the clinic, using an “ecological” approach that allows providers to understand patients’ behavior and mood in patients’ natural environments. Combining these data with remotely-administered self-report surveys (“ecological momentary assessments” or EMAs) and standardized clinical assessments may improve providers’ ability to detect certain clinical conditions, assess their severity, and guide personalized treatment recommendations [11–13].

While clinical inference is the primary goal for using CP technologies in care, these tools can also lead to incidental or secondary observations that may be sensitive and not yet consented to. Ethicists have raised concerns about patient privacy and the need for informed consent to the use of such sensitive data [14–16]. However, it remains unclear what patients need or want to know about AI-based CP tools, including their potential impacts on privacy and care quality, to provide meaningful consent [17, 18]. Understanding patients’ informational needs is critical for developing targeted education and consent procedures that address their concerns [19–21]. As we argue elsewhere [22, 23], these understandings can support participatory approaches to co-designing treatment plans that enhance patient engagement, autonomy and empowerment [24, 25]. This paper aims to sensitize researchers and practitioners about what patients and caregivers need to know to meaningfully consent to the use of CP technologies in clinical care.

## Background

Little is known about the range of patients’ and caregivers’ questions and concerns regarding the use of AI-based technologies using CP data in clinical care. Approaches like digital phenotyping and computational behavioral analysis combine digital sensors with algorithms to analyze vast amounts of data [26]. This technological complexity and data density, as well as the nature of data collection in both clinical and ecological settings, introduce questions about how (and what) to communicate to patients about risks and benefits. Beyond concerns around privacy highlighted in the existing literature [14, 27, 28], many patients may not fully understand what data are collected, what inferences may be made, how data are stored, used or shared, and other ethical and practical considerations that may impact patients’ informed consent decisions [15].

In their “ethics checklist” designed to help researchers establish procedural safeguards around digital phenotyping, Shen et al. [29] include three key questions related to informed consent, including whether researchers have 1) “appropriately adapted [their] informed consent procedures to [the] specific study population, including possible use of surrogate consent”; 2) provided “background education on relevant technologies, such as explaining what social media companies may already be doing with the participant’s data”; and 3) “determined what a reasonable person would want to know, and explained in [their] IRB proposal the evidence on which [they] reached that determination.” To date, there is limited empirical data to provide robust answers to answer these questions, leaving gaps in understanding patients’ and caregivers’ informational needs when considering the use of digital phenotyping or related approaches in their care. It remains unclear how consent procedures should be tailored different stakeholders, considering factors like age, role (e.g., patients versus surrogate decision-makers), socioeconomic status, and other sociodemographic variables. Effective informed consent requires understanding patients’ and caregivers’ knowledge gaps about the purpose and impacts of these technologies [30]. This paper presents insights from interviews with adolescents and caregivers, offering an overview of their informational needs to help researchers and providers develop educational strategies that respect patient autonomy and enhance patient engagement in using CP technologies in ways that align with treatment goals.

## Methods

As part of 4-year study funded by the National Center for Advancing Translational Sciences (R01TR004243), we conducted in-depth, semi-structured interviews with adolescent (*n* = 20) and caregiver (*n* = 20) dyads to explore their perspectives on potential benefits, risks and concerns around the integration of CP technologies into their care. Respondents were recruited from a “sister” study (5R01MH125958) aiming to validate CP tools designed to quantify objective digital biobehavioral markers of socio-emotional functioning.

### Participants

Participants included a clinical sample of adolescents (aged 12–17 years) with varied diagnoses, including autism, Tourette’s, anxiety, obsessive-compulsive disorder, and Attention Deficit/Hyperactivity Disorder (ADHD), as well as their caregivers (typically biological parents, Table 1). Diagnostic presentations for all adolescents were confirmed by expert providers using a battery of established clinical measures. Adolescent-caregiver dyads were referred to the current study by the sister study’s coordinator and then contacted by a research assistant (AS) via phone or email to schedule an interview. Participants were interviewed between January 2023 and August 2023.

**Table 1:** Demographics for Interviewed Adolescents and Caregivers.

		Adolescents		Caregivers		TOTAL
		*n* = 20	% Total	*n* = 20	% Total	
			50%		50%	*n* = 40
Gender	**Male**	12	30%	2	5%	35%
	**Female**	8	20%	18	45%	65%
Ethnicity	**American Indian or Alaska Native**	0	0%	1	3%	3%
	**Asian**	1	3%	1	3%	5%
	**AA/Black**	5	13%	4	10%	23%
	**Native Hawaiian or Other Pacific Islander**	0	0%	0	0%	0%
	**White**	17	43%	15	37%	80%
	**Hispanic or Latino**	4	10%	2	5%	15%
	**Not Hispanic or Latino**	16	40%	18	45%	85%
Marital Status	**Married and living with spouse**	-	-	13	65%	65%
	**Widowed**	-	-	1	5%	5%
	**Divorced**	-	-	4	20%	20%
	**Separated**	-	-	1	5%	5%
	**Never Married**	-	-	1	5%	5%
	**High school only or less**	-	-	0	0%	0%
	**Trade school/Associate’s degree**	-	-	2	10%	10%
	**Bachelor’s degree**	-	-	10	50%	50%
	**Master’s degree**	-	-	4	20%	20%
	**Doctoral degree**	-	-	4	20%	20%
Parental Status	**Biological Parent**	-	-	18	90%	90%
	**Step Parent**	-	-	0	0%	0%
	**Adoptive Parent**	-	-	2	10%	10%
Diagnosed Condition	**OCD**	4	20%	-	-	20%
	**Autism**	5	25%	-	-	25%
	**ADHD**	3	15%	-	-	15%
	**Anxiety**	4 (1 self- reported)	20%	-	-	20%
	**Tourette’s**	1	5%	-	-	5%
	**No clinical diagnosis or symptoms**	9	45%	-	-	40%
	**Average age**	14.9	(s.d. 2.2)	48.3	(s.d. 6.4)	

*Values may not total to 100% due to categorical overlap (e.g., comorbidities).

### Data collection

Separate but parallel interview guides were developed for adolescents and their caregivers, with the same constructs explored across both stakeholder groups (KK-Q, AS, MH), including: perceived benefits and concerns regarding integrating CP tools into clinical care, impacts on care, attitudes towards automatic and passive detection of emotional and behavioral states, perceived accuracy and potential for misinterpretation/-attribution/-classification of symptoms or conditions, clinical utility and actionability, data security and privacy concerns, potential for unintended uses, perceived generalizability and potential for bias. These domains were chosen based on issues raised in the clinical and ethics literatures (see Background) and with the guidance of experienced bioethicists and child mental health experts. Initial drafts of the interview guides were piloted with two psychologists (ES, CJZ) specializing in adolescent mental health, resulting in minor clarifications in wording. Interviews were conducted via a secure video conferencing platform (Zoom for Healthcare) and lasted an average of ~45 min (AS). This study was reviewed and approved by the Baylor College of Medicine Institutional Review Board (H-52227), which waived a requirement for written consent; thus, participants provided verbal consent.

### Data analysis

Interviews were audio-recorded, transcribed verbatim, and analyzed using MAXQDA software. Led by a qualitative methods expert (KK-Q), team members (AS, MH) developed a codebook to identify thematic patterns in adolescent and caregiver responses to questions addressing the topics above. Each interview was coded by merging work from two separate coders (AS, MH) to reduce interpretability bias and enhance reliability. We used Thematic Content Analysis [31] to inductively identify themes by progressively abstracting relevant quotes, a process that entails reading every quotation to which a given code was attributed, paraphrasing each quotation (primary abstraction) and further identifying which constructs were addressed by each quotation (secondary abstraction), and organizing constructs into themes. To enhance the validity of our findings, all abstractions were validated by at least one other member of the research team. We then calculated the number of respondents who discussed each theme to obtain a sense of the proportion of stakeholders raising or discussing each concern/theme. In rare cases where abstractions reflected different interpretations, members of our research team met to reach consensus. Frequencies and percentages are reported as descriptive rather than inferential statistics and are therefore not intended to suggest any level of statistical significance.

## Results

In order of salience (frequency discussed, see Methods), adolescents and caregivers highlighted questions and concerns across seven domains, including 1) Clinical Utility and Value; 2) Evidence, Explainability, Evaluation and Contestation; 3) Accuracy and Trustworthiness; 4) Data Security, Privacy and Misuse; 5) Patient Consent, Control and Autonomy; 6) Physician-Patient Roles and Relationship; and 7) Patient Safety, Security, Well-being and Dignity. While both adolescents and caregivers expressed informational needs and concerns across these domains, we observed the greatest differences between adolescents and caregivers in relation to caregivers’ greater emphasis (75%) than adolescents’ (35%) on the importance of ensuring informed consent, control and autonomy over which data are collected and how data are integrated into care (See Table 2). Caregivers (95%) also had more questions than adolescents (65%) about the clinical utility and value of CP for care. Adolescents (90%), on the other hand, expressed greater desire than caregivers (60%) about knowing why/how certain inferences are made from their data and wished to preserve the capacity to contest them in cases where they may contradict adolescents’ own subjective experiences. Details are discussed in turn below and relevant quotations illustrating each theme are presented in Tables 3–9.

**Table 2:** Observed Frequencies of Stakeholders’ Informational Needs about Integrating Computer Perception into Clinical Care.

Informational Need Domain	Adolescents (*n* = 20)		Caregivers (*n* = 20)		TOTAL (*n* = 40)	
Clinical Utility and Value	13	65%	19	95%	32	80%
Evidence, Explainability, Evaluation and Contestation	18	90%	12	60%	30	75%
Accuracy and Trustworthiness of CP	12	60%	16	80%	28	70%
Data Security, Privacy, and Misuse	12	60%	15	75%	27	68%
Patient Consent, Control, and Autonomy	7	35%	15	75%	22	55%
Physician-Patient Relationship and Role	11	55%	12	60%	23	58%
Patient Safety, Well-being and Dignity	5	25%	10	50%	15	38%

Frequencies were calculated by counting all unique respondents who raised/endorsed each of the subtheme concerns (see 3. Methods). Frequencies and percentages reflect only reported information and are not intended to suggest any level of statistical significance.

**Table 3:** Questions about Clinical Utility and Value.

Questions about benefits, types of feedback to expect, customization of data inferences, and capacity to detect current and future disorder.		
	Patient Perspectives	Caregiver Perspectives
Value Add	[Is this a] more effective way of diagnosing me with problems? [Will it] make it easier on everyone? P11	I think it’s fine if they use it. I would just want to know why they’re using it. What is the purpose? What are you looking for?… Because I’m not going to let you do it for nothing. CG08
Informational Gains	If you’re able to find out how [the tools] know that from the certain feelings you’re displaying, then I feel like it would make me more aware… If there’s something about the way I’m acting that… I could be aware of, I feel like I would care more about that. P14	I think if the studies show that they’re useful, then if you share that with the parents, from our studies we’ve seen that this data indicates this, then that would be useful. I mean, obviously this is very specialized, and so if it’s just given to the parents without any explanation or elaboration, then it may be limited in their use. But I think if you can show us that studies indicate that if data is like this, then this is the situation and this is how we recommend you treat it or respond, then that would be very helpful. CG02
Personalized Relevance	I think the heart rate and stuff like that could be interesting, and my voice, but stuff like actual emotions, I don’t know. Because sometimes those change really fast and I feel different. I don’t know. I don’t know if it would be able to pick up on that as well as it thinks it can. P14	It does sound useful and beneficial, but I do wonder how it would help with an autistic who’s already overwhelmed because then there tends to be no facial expression and sometimes even the voice turns monotone. CG09
Augmentive Capacity	Just because with a physical problem you know already what’s happening, but with mental stuff, I mean you can’t really look into your brain and see what’s going on. So it would help for that. P13	I’m [an] overly anxious patient or person in general, and I’m also very analytical, I would probably inquire a moderate level of detail to be provided. I would actually want to see some key results, I suppose, some diagnostic elements that led to that conclusion.And I don’t think that I have the proper education to go in further detail and ask for the full analysis of the data elements and all that backend stuff. Some perimeters, I would feel much better having, just a name, a label with a diagnostic being presented to me by my MD. CG03
Actionability	Just because I feel like if you’re able to find out how they know that from the certain feelings you’re displaying and stuff, then I feel like it would make me more aware. But if it’s just something physical, then it’s like that’s all there is to do about it. But if there’s something about the way I’m acting that not I could change, but I could be aware of, I would care more about that. P14	I think if the studies show that they’re useful… that studies indicate that if data is like this, then this is the situation and this is how we recommend you treat it or respond, then that would be very helpful. CG02

**Table 4:** Questions about Evidence, Explainability, Evaluation and Contestation.

Questions about algorithmic explainability, types of evidence available, how physicians confirm algorithmic conclusions, right to redress/contestation		
	Patient Perspectives	Caregiver Perspectives
Explainability	… with ChatGPT and all those types of algorithms…. if you don’t know too much about them, it can be really intimidating. So having a good way to figure out, “Oh, so this is how my data’s being used, this is how patterns are being made, so I can get this diagnosis.”… that would be really comforting for me, for a lot of people, I’m pretty sure too. P16	Because I’m, I guess, overly anxious patient or person in general, and I’m also very analytical, I would probably inquire a moderate level of detail to be provided. [If] I am being observed by different sensors and then come up with she’s bipolar with ADHD and whatever other diagnostics, I would not be completely comfortable with just that. I would actually want to see some key results… some diagnostic elements that led to that conclusion… I don’t think that I have the proper education to go in further detail and ask for the full analysis of the data elements and all that backend stuff. CG03
Evidence / Validity	Because they’re new, I would probably ask questions because data hasn’t been collected that much. So there is always that with new software, it’s like, “Oh, how much is this actually trustworthy? How much is it actually truthfully picking up?” But after a while, I think even a year from now I’d be like, “Oh, well, this person seemed to have a good experience with this device being able to track and help them out, and it actually was able to track, okay, they might actually be in a depressed phase, that they could possibly go into a deeper condition when normally if we didn’t have this device or this technology, it wouldn’t be able to track that, and by that point it would already be too late.” P19	I would want to know as far as trial, was it a trial based thing? How long has it been out? How many patients did they test it on? What was the procedure? What makes the doctor feel that this is the way to go at this day and time? What has them thinking that they could rely on the information? CG06
Interpretability	[Would want to see data]… maybe if the technology would be able to ask you questions about, Hey, we tracked this, can you explain why it could have tracked this? And you could have the option to say, “I don’t know, it’s probably just a fluke,” or actually explain it. P19	I’ll take all of it. I will take it all. Give me, I might not even understand the raw, but I’m going to figure it out. I will figure it out. And if you explain it to me and say, “Okay, well this is here, you see this and this line and this points.” Okay, now talk to me in regular people language and not doctor language or computer language., I’m very much about, prove it to me. Show me. Just because this state it, don’t mean I’m going to believe it. You’ve got to show me how and why you came up with this. CG08
Verification	Just why and if there were any errors in it, if there’s a way that that might not be true or something… because you probably don’t want that. P15	If the doctor could not provide any information to support the diagnosis other than [what] the computer says, then I would be a little bit uncomfortable with that. But if the doctor was like, we’ve noticed she’s showing these symptoms on the computer and then I know this also happened … I would want the doctor to feel confident about the diagnosis and be able to demonstrate why he or she thought that was a correct diagnosis as well. CG17
Contestability	Yeah, it would be nice to know how it came to that conclusion because sometimes some algorithms are just like, “Based off of these patterns, I’m going to produce this output,” which might not even be true. So if there was a way to see, “Oh, this is how… It picked up on these patterns.” And then you can double-check with the person and be like, “Oh no, I actually don’t do these things. I don’t know why it produced that.” It would be helpful, especially for misdiagnosis, to litigate, I guess. P16	Okay, give me what you got there. Explain to me what are we looking for? What are the symptoms? What are the treatments? Give me the whole information and have it… I would want to know that they had it in their heads that, “Well, we need to verify this.” So if there was a verification, what is it?

**Table 5:** Questions about Accuracy and Trustworthiness of CP.

Questions about the trustworthiness of CP tools, generalizability across conditions, algorithmic bias, accounting for context and subjectivity, methods for evaluating trustworthiness		
	Patient Perspectives	Caregiver Perspectives
Inference Capacity	I’d ask to see how they got that or what information made them think that. And then, I don’t know, it depends what disorder it is, but yeah, I just want to see how they got that. P18	I would want to know… what data points are being collected … If it’s noticing some sort of increase in a breathing rate, it could mean A, it could mean B, I would kind of want to know what the potential limitations are to some of those things. I guess the algorithms are probably huge and numerous and super interconnected, but how the pieces come together to form a diagnosis? If I’m sitting around for two days straight, that doesn’t necessarily mean I’m depressed, but if I’m sitting around for two days straight with this and this and this, that might be more of a flag. So I kind of want to know what pieces get connected to that to make that determination. CG10
Physician Reliance	Maybe if something in particular happened that made you sad for a period of time but it’s not permanent, then I know that I wouldn’t want that to be taken out of proportions and maybe it would become a thing even if I didn’t want it to, it was just something temporary. P14	I don’t like the idea of deciding whether I’m happy or sad, perhaps, based on … data coming from my phone or having a doctor saying, “Oh, she’s using the phone this much.”… Well, I’m listening to podcasts, I’m looking things up, I’m doing my financial stuff. And so, just thinking that I could be sitting in a chart and prejudicing or giving a bias toward a provider that could impact the care that I would receive, I don’t like the idea of that. CG12
Accuracy	Because they’re new, I would probably ask questions because data hasn’t been collected that much. So there is always that with new software, it’s like, “Oh, how much is this actually trustworthy? How much is it actually truthfully picking up?” P19	The data sometimes can be manipulated sometimes but it’s just ensuring the data integrity, knowing that it is all collected with good intent, and being presented accurately. I work with data and I understand you can tell a different story based on the data that you have. Having that dialog still with the physician, possibly looking at another test, talking with [redacted] too. CG12
Potential for Bias	I’d want to know that it’s tested equally on both men and women and across racial groups and ages and all different factors. P07	The second concern, it’s more about just the technology… and all the issues that have been identified with, for instance, differences in races. So some facial recognition systems do not work with people of color, right? So, how do you make sure that they’re fair? They treat people, women, men, people of color, different ethnicities, culture, all those different things that make a person what they are. They might not be captured by those facial recognitions. So they might be warning a doctor like, “Oh, this person is depressed,” but it’s just because they’re are Latino, or they’re white or whatever. Or maybe they’re just Northern European and they don’t smile as much as Latin Americans, which is what the data was trained with. CG15
Participatory Development	Sometimes really with the development of the AI and the code… having a diverse group of coders and people working on that could really destigmatize… that part, but there is always some sort of bias somewhere, but hopefully it won’t make a big impact on any sort of groups. So just by having a diverse group of people working on the project… I don’t think there should be that much worry about bias. P16	I appreciate the fact that people want to know how [this technology] is going to affect people. I would definitely say that if I feel that it is looked at by the human rights people, the people that make the laws on HIPAA, the big boys. But I would want them to do it together because if you independently look at it, you’re going to get a different view. They got to sit them down, they got to powwow it, they got to get some people on the board that are Joe Smoes, whatever, and they got to do the right thing. Traditionally, we haven’t quite seen that in all areas. CG11
Accounts for lived experience	I think for the most part I’d be fine with it, but if it’s constantly picking up on the five minutes that I just didn’t get exactly what I wanted and it would just be like, “Oh my gosh, you’re not feeling okay, you are possibly depressed,” and it’s like, “No, it was just a five minute thing.” I feel like it could eventually get to the point where it’s annoying, but I would be fine with it just sending a message at the end of the day to a doctor and just being like, “Here’s a list of things, list of possible emotions that this person was having.” P19	But with children in particular, they are not so much trained socially. What do you call? They don’t have responses or they would not exhibit symptoms. They are are socially trained or formed. It might be the variety of the symptoms and then the variety of the emotions that may be hidden behind those that could add a layer of complexity to how the technology would actually be able to diagnose. CG03

**Table 6:** Questions about Data Security, Privacy and Misuse.

Concerns about the duration and type of data collection, data access and portability, data sharing and protection, linkage with other personal data, legal ramifications		
	Patient Perspectives	Caregiver Perspectives
Data Quantity	I don’t think I would be very opposed to it, and I would consider it and talk to my parents and stuff. And yeah. It depends on what it requires out of me. Do I have to spend a long time recording my movements and yeah. P03	I would want to know what they were collecting and for how long. If they’re going to do it for six weeks, I would want to know some assurance that at six weeks it’s no longer collecting data. And then how they were going to protect the information. CG17
Data Types	I would probably want to know exactly what they were tracking, my feelings or what I’m doing, where I’m going and stuff like that. P20	I’d want to know exactly what information it’s gathering, when it’s gathering it, what it’s tracking. CG07
Data Access	I would just want to know that my information was private and it was only going to be shared with my doctor and no one else. And that whatever information that my doctor got from the tracking that I would have the freedom to look at it as well. P17	I would want the privacy of everything to be kept in my own control or [my child’s] own control once she’s an adult so that it’s not used against her, because we’ve had that happen. CG05
Intended Data Uses	I think I would definitely want to know what information is being tracked and how or where the information is going and how it’s being used, I guess. P02	Well, I think with that, it should be disclosed if that’s the case so at that moment, I can say, “Oh yeah, they’re going to use this for future research.” Then I’m totally fine with it. But I don’t think it should be a surprise thing. Oh, you thought it was for the doctor, but no, it’s being used for other stuff. I think that should be disclosed, and then I wouldn’t have a problem with it. CG06
Data Protection	Yes, because if I lose it somewhere, then other people might be able to see it maybe … I would want it to be in a private spot where it needs a code or something. P10	But as far as being stigmatized, I’d want to know that the data, I’d want to know exactly who has access to the data and that nobody else can have access to the data without me giving my permission. And then, like I said earlier, I’d want to know exactly what the data was meaning is it just a tone of voice or, heaven forbid, this doesn’t even impact me. But as I think about things out there, so a custody battle’s going on for a kid, can they call in, is it actually recording stuff that’s going to have and be used against you? To me, all of that should be very transparent. CG12
Data Triangulation & Inference	I guess how the computer tracks everything. How personal does it go into? Does it only just track using my phone or does it go into my computer? All sorts of things like that. How much does it go forward? That would probably just be my main concern and also just about privacy information, how it’s kept and such and who reviews it. P16	I guess I want to know where is that data being stored that has access to every conversation he’s having. Is it picking up everybody around him talking? Who can access that? Can the doctor themselves go in and listen to a conversation because I wouldn’t like that. Now, if the data’s being analyzed in a way that the doctor doesn’t have access to and it’s shooting off reports, I’m far less troubled by that. CG12
Unwanted Use of Data	Yes, mainly just information breaches, especially of personal, private information. I probably really don’t want a lot of people to know about it, especially if it’s like, “Oh, I have anxiety,” or “I have depression,” or something like that. And that being shared is a big breach of privacy, so that would be a main concern of mine if computer perception is being used. P16	I’m just trusting that that data’s not going to be used to violate my privacy. So, I don’t know, I would just want some reassurances about what the data can be used for, I want an assurance that it could never be used against you legally. I can’t even think of what I’d be doing though that I wouldn’t want a law enforcement officer to know, but I don’t know. I would want to have a very good idea of what the limits of its usage were, but I do think it could be useful for mood. CG12
Data Sharing Decisions	For myself to use that, I think I’d have to know that it would show me the results first and then ask if I’m okay with sending it to the doctor. Instead of just sending it to the doctor first and then the doctor telling the things. P07	Any thoughts or ideas that I wouldn’t want to go to his doctors. No, not in the construct that I think you explained where it’s going to be generalities. Now, again, because I would want my own privacy protected, I wouldn’t want, again, the minute details to be able to be playable to the provider without my explicit consent. And I think I stated already I wouldn’t want a provider to be using that data to exclude what I’m telling them there in that moment to the point of you come in and you’re very concerned, let’s just pick on something like pain. CG12
Data Exchange Transparency	I mean, I guess I would want to know what they’re taking, what specifically are they taking, I guess kind of like a contract or something. Or when I’m making a deal with someone, I want to know what are the standards or what is the issue, I guess, what are they trying to measure? Stuff like that, I’d want to know. P01	I need to know what are you going to do with that? Who are you going to share that with? How are you going to document? So… if this all comes about, where they really do institute higher levels [of surveillance and inference] than the Fitbit, they’re going to have to define that through HIPAA. You see what I mean?… [And] when you talk emotion, behavior, that’s mental health. That has to be exclusive and there’s going to have to be permissions there. [I would say] “No, you may not. You can monitor this. You can monitor my nervous system, you can monitor my heart, my lungs, those things. But when it comes down to my emotions, those are mine.” CG11.

**Table 7:** Questions about Patient Consent, Control and Autonomy.

Questions about transparency around data collection, patient and guardian rights, consent, control over data collection and feedback		
	Patient Perspectives	Caregiver Perspectives
Data Collection: transparency	I mean, I guess I would want to know what they’re taking, what specifically are they taking, I guess kind of like a contract or something. Or when I’m making a deal with someone, I want to know what are the standards or what is the issue, I guess, what are they trying to measure? Stuff like that, I’d want to know. P01	Just like what is the right level of access to your doctor? There’s some information, health information that I’d be happy to share, my heart rate or my oxygen level if the app measures that or how many steps I do on a daily basis, the average, that type of thing. I think that’s fine. Other more invasive information, it’s like the messages or the GPS location. I don’t share the GPS location with my mother, let alone with my doctor. So that feels a bit overly invasive. I understand at least the principle of how it could be used for depression or certain things, but no, I wouldn’t be willing to share those. CG15
Right to Refusal	Because I can revoke, or grant, because I should be able to revoke, or grant access to certain information. And in certain systems it’s actually required to be able to revoke, or grant access, by law. P09	I think either potentially the ability to change as time goes on and maybe you see how this data’s being used and are there benefits to sharing and fewer risks to sharing than you originally anticipated? Or as things are learned or discovered about the data, and maybe more confidence about what the data, where it goes and what it is used for, then there may be more comfort in bringing up some of those restrictions. CG01
Control: Transparency	I think the main thing that would make me more comfortable with using it is that I get to approve who the data goes out to, the specific doctor’s office and things like that. P07	I’d want to know that the data, I’d want to know exactly who has access to the data and that nobody else can have access to the data without me giving my permission. And then, like I said earlier, I’d want to know exactly what the data was meaning is it just a tone of voice … and if we have any incidents of medical providers taking advantage of that trust relationship and having data that the patient’s not aware of or access to it, I think it’s very, very bad for AI. So, I’d want to make sure all studies took that so seriously that a court order couldn’t give them access to data that I’m unaware is even being taken. CG12
Accessibility & Ownership	I wouldn’t care if it was for a good cause, a research study or some medical study. But if it was for a company, then yeah, I’d like some money from [the use of my data]. Maybe if there’s a product that they’re making with my data, I’d get a discount on that product or something like that. P07	I would expect there would be an alert, some notification that that is happening. And I would probably even check it once a week anyway myself, kind of like what I do with my bank accounts, just go check and see if the charges are what I made. That’s something that I would feel comfortable with. If it were to be once a month, then I wouldn’t be able to see if maybe someone accessed the data that I didn’t know was going to access the data or I didn’t have an understanding and I would be able to reach out that much sooner to the research institute and start asking questions. CG16
Control of Feedback	It’s just personal information and having some control over who sees it is reassuring because it’s not going out to everyone who I don’t want my information passed to everywhere or given to everyone. P16	…if she knew, for example, it was recording her sleep patterns, I think it would make her anxious at bedtime….[and] because she’s a minor, I probably wouldn’t tell her everything it was tracking. I wouldn’t lie to her, but I may not just give her as a parent all the details because I wouldn’t want her to worry about it. CG17
Alerts and Notification	I don’t think I would say it I don’t want to do it one and done. I would maybe change it. Because I mean, right now I have all these opinions and how I may be feeling now is probably going to be different from 20, 30 years from now. I may not trust the same people. I mean, things happen in our lives every day and things… One day you’re fine and the next you’re not. I think it’s definitely something… I wouldn’t just say, oh yeah, I trust these people now I’m done. No I’d want to probably change at some point. P01	I would only be comfortable with this type of information being shared with the physician and for treatment purposes…. But if… I don’t know, not my son in particular, but what if someone exhibits suicidal behaviors or pre-cursors of any type of suicidal, or certain type of distress that would make him a serial killer? Would I want that information to be shared just with the physician? Probably not. I would probably want any type of authorities at some point be alerted as well, because that puts not only the person but some others at risk from a safety perspective. CG03

**Table 8:** Questions about Provider-Patient Relationship and Role.

Concerns around preserving patient-centered care, impacts on the patient-doctor relationship, strategies for conflict resolution		
	Patient Perspectives	Caregiver Perspectives
Patient- Centered Care	I don’t know if I would feel as comfortable hearing [the results] straight from a computer rather than my doctor. I would feel more comfortable it coming from a person who can explain it to me better than just a computer. Because I think that humans can relate to me and if it’s someone talking to me and like I feel more comfortable when someone can like, I know that there’s things that they know that a computer doesn’t know about me, like human situations and they can talk to me about that stuff. But a computer would just give me an answer. P14	I wouldn’t want a provider to be using that data to exclude what I’m telling them there in that moment to the point of you come in and you’re very concerned, let’s just pick on something like pain. So, if you went in to your doctor and said, “I’ve been in so much pain every day,” and then they have data that’s like, “Well, but you’re doing everything like normal so she must not be in pain, I’m not really listening.” If I had a serious concern about my son, I wouldn’t want a doctor to be biased against my concern because the data didn’t seem to show anything. Because I think that a parent’s observations should be every bit as important as the data, if not, more depending on what exactly it is we’re talking about. So, I think I would want it used with great caution, possibly a supplement. CG12
Physician-Pt Relationship	I’d rather see [the data] from the doctor … because I feel like there’s more of an emotional thing than with just a computer showing me the raw results … I’d still prefer to have a face-to-face discussion with a doctor, another human to tell me it. I feel like there’s an emotional part that computers just can’t replicate. P07	[Both] technology and humans are behind the design of this, and so I consider more of my concern being … Hopefully, not replacing a human that we need to connect with. I feel that despite all the technology, that we still have to make human connection, so that’s where my concerns are, loss of human connection… CG16
Prioritize Subjective Experiences	As doctors or researchers, I guess just to keep me in the loop. I mean not every day, not every week necessarily, but … If something pops up or something doesn’t go right, I want to know. I don’t want them to try and put it off or think, oh, okay, this isn’t important to me as a scientist and probably the patient or the person whose information is… They probably think the same thing that I’m thinking. I want them just to loop me in. I don’t want them to just hide or discard any information that they don’t think is important. P01	On the other hand, if the data’s trying to show me that, I don’t know, my son has a personality disorder or something I’ve never seen any manifestation of that, I wouldn’t mind questions about it but I would hope that it would be quickly eliminated or they would be able to provide me a lot of information as to why. C12

**Table 9:** Questions about Patient Well-Being, Safety and Dignity.

Questions about regulatory oversight, customization of patient monitoring, and stigma/dignity concerns, possible negative impacts		
	Patient Perspectives	Caregiver Perspectives
Safety Regulations	It really depends on who it’s getting shared to, but I guess it would be the same as what HIPAA is for. It would just be sent to the people that would be able to act upon it super fast and people who wouldn’t have an opinion on it. P19	They’re going to have to come up with some info. Give me some background. Who’s the company that designed it? How long has it been studied or whatever before it hit? Is it an authorized FDA? Because FDA’s got to authorize anything that’s used for medical, whether it’s machinery or whether it’s medicine. So I would want to know those things and will I actually have the right to refuse? CG11
Customization of Monitoring	I’m a very type A person. Go, go, go. Even if you stay home for a while or don’t get out of bed and things, [or] even if I’m really stressed or having a bad day, I don’t do that because I’ll always have a to-do list, even if I’m having a bad day, I have to do these things. It [the algorithm] might not realize sometimes that I’m overly stressed or upset just because I’m still going on with my normal day. P17	Let’s say, [it] was tracking her phone and it had rained. She loves to go running. That’s her outlet, to go running outside. Well, sometimes in the summer months we can get rain every single day in southeast [state]. So if it rains every single day and she can’t go running, it might be a misleading piece of information that would suggest that maybe she was depressed when in fact she wasn’t ever depressed. She had OCD. Does that make sense? CG17
Potential Stigma	I know what it does, but what am I wearing? What are they making me wear or what are they putting on me? When I can take it off … when it’s used. P18	Also probably the stigma attached to her if it’s something that is going to draw attention to her and make her feel uncomfortable, I would obviously want to rethink that. But if it’s not harmful and it doesn’t draw attention to her and she was okay with it, I don’t see myself not consenting. CG10
Potential Discrimination	I think race is probably a factor. I don’t know that much about it. But I feel like it could easily become something where it can marginalize a group and not give a certain group of people the right care because it misunderstands something or it’s created by a certain race of people. And then, it only applies to that certain race of people or things like that. Or gender might play a role in it. If it’s created by men, then women may not be able to use it as efficiently. P07	I just worry that it could be wrong because sometimes computers don’t know everything. They’re not human. And it would be messed up for it to say, oh, well this person is feeling this and that person is not feeling that. And then it could mess up something. Oh, your child’s feeling sad, to the point where they want to kill themselves. And they’re like, “No, that’s not what I feel.” And so now that’s bringing another layer in to something. So I just worry about the error rate. That would be something that I would concern myself about. CG08

### Clinical utility and value

Nearly all caregivers (95%) and most adolescent (65%) expressed a need for information about the clinical utility and value of integrating CP tools into care. Eight adolescents (40%) and 15 caregivers (75%) wanted to know the tangible improvements and justifications for using CP in their care, and actionability of CP in influencing treatment direction. Around half of the caregivers sought specific justifications for using of CP in their child’s care in order to understand the clinical utility and wanted to know how providers gauge the clinical significance of the data being collected. Six adolescents wanted to know whether CP tools may help them gain greater self-awareness of their symptoms and unconscious behaviors. Both groups had questions about CP’s potential to improve provider’s ability to detect or predict future disorders. Overall, the informational needs in this domain focus on understanding CP’s tangible benefits, expected feedback, and the clinical rationale behind data collection.

### Evidence, explainability, evaluation and contestation

Almost all adolescents (90%) and over half of caregivers (60%) raised questions about how clinical inferences are made from CP data and wanted the ability to challenge data that might contradict a patient’s subjective experience. Just over half of caregivers (11) and adolescents (six) suggested that a basic understanding of CP algorithms could help assess accuracy and trustworthiness, and contest inferences they disagree with. Seven respondents wondered whether providers would explain how CP algorithms derive inferences (e.g., what pieces of information are used to classify diagnosis) and what evidence would support their validity. One caregiver emphasized the importance of seeking confirmatory evidence before making significant clinical decisions based on CP outputs. However, 12 caregivers and nine adolescents expressed that they did not necessarily need to understand the algorithm’s workings, trusting their provider’s judgment instead. These findings illustrate the need for information on the trustworthiness and explainability of CP algorithms, available corroborating evidence, and preserving the right to contest algorithmic conclusions.

### Accuracy and trustworthiness of CP

Over half of adolescents and 17 caregivers expressed informational needs regarding the accuracy, clinical validity, bias, and relevance of CP tools in healthcare. A few adolescents (three) wanted details about the quality and inclusivity of training data sets – specifically the distributions of race/ethnicity, age and gender – before consenting to CP use in their care. Around half of caregivers sought similar information, including success rate data from validation studies, the developmental phase of the technologies, and whether patient perspectives were considered during development. Both groups also wanted to know how contextual factors, such as weather, schoolwork, socioeconomic circumstances or environmental insecurity, are accounted for in data interpretation.

### Data security, privacy, misuse

A majority (60%) of adolescents and (75%) caregivers had informational needs related to what types and how much data would be collected, who might access or use their data, how it will be protected against unwanted uses or triangulation with other personal data, and how patients will be safeguarded from stigma or discrimination based on the data collected. About a third of participants (eight caregivers and five patients) emphasized the importance of data privacy, with some viewing CP data as more sensitive and deserving of greater protection and security than other physiological data (e.g. heart rate, blood glucose, etc.). Over half of all caregivers and adolescents expressed concern over potential unintended uses of their data and sought assurances that using CP tools would not negatively impact the patient now or in the future. Caregivers in particular, worried about stigma and discrimination if the data were accessible to future schools, employers or the justice system. Some patients preferred that privacy guidelines be clearly communicated early in the consent process to ensure informed consent when using CP.

### Patient consent, control and autonomy

Three quarters (75%) of caregivers and approximately a third (35%) of adolescents highlighted concerns about transparency in data collection and their rights to participate in decisions about using CP tools in care. Both groups wanted to be informed of when CP would be used in their care and questioned whether they would be afforded a choice over which data are collected, which feedback is returned and whether they will have access to data, inferences and summaries. Preferences varied, with some wanting full access to the patient’s raw data to track changes and identify patterns, while others preferred to receive only summaries or alerts of significant changes. Some adolescents (five) and caregivers (six) wanted to know how involved they would be in decisions regarding data disclosure to providers. Certain adolescents (five) viewed themselves as owners of their data, wanting the right to directly grant or refuse access. Around three-quarters of caregivers and adolescents (13 adolescents, 16 caregivers) expressed a desire to restrict data access to providers and research teams, excluding corporations. Others (five adolescents and six caregivers) wanted the capacity to vet which information is shared, preferring that device interfaces share results with patients first, asking permission before sending data to providers. Over half of all respondents (14 adolescents and 11 caregivers) were concerned about being able to turn off passive monitoring in intimate, private or atypical settings.

### Physician-patient relationship / Role of a physician while employing CP

A total of 11 adolescents (55%) and 12 caregivers (60%) had questions about the role CP data would play in their clinical care, how providers would ensure that personal experiences remain prioritized in CP-guided care, and how CP tools might impact their interactions with providers, especially when CP contradicts patients’ self-reported experiences. Some respondents (seven caregivers and three adolescents) raised concerns that providers may over-rely on data in clinical decision-making and sought assurances that patients’ subjective experiences would still be central to care. One caregiver suggested that while CP data should be considered, providers should use CP outputs to inform questions and discussion points with patients, rather than stand in for their perspectives. Five respondents (two caregivers and three adolescents) expressed concerns about CP tools disrupting daily life, and six caregivers felt that CP use might over-extend the provider-patient relationship, potentially crossing boundaries if providers could automatically detect a patient’s emotions. These informational needs address the preservation of patient-centered care in a data-driven environment, ensuring that patients feel heard, seen and autonomous.

### Patient safety, well-being and dignity

Five adolescents and ten caregivers wanted to know what measures are in place to ensure CP technologies are officially sanctioned, respect diverse expressions and experiences of distress, and maintain patients’ dignity in social settings. A small number of caregivers (four) shared that they would like to use CP as a method to prioritize patient safety by becoming aware when their child might be putting themselves in danger during a mental health crisis. Another suggested that CP might help to ensure clinical attention for patients who may not otherwise be able or willing to communicate when they are experiencing anxiety, sadness, and depression. On the other hand, some adolescents expressed that this must be balanced with allowing adolescents to deal with their emotions independently, when appropriate, and not using CP as an additional means of parental monitoring. In addition to protecting patient safety, some caregivers (four) also expressed wanting to preserve their child’s dignity and prevent stigma through wearables. Some worried about CP devices drawing unwanted attention to patients in ways that may be experienced as stigmatizing, particularly among adolescents undergoing numerous developmental and social changes.

## Discussion

This paper aims to inform providers and researchers about the informational needs and preferences of adolescent patients and their caregivers regarding using CP tools in their care. While ethical discussions to date have focused on data security, privacy and misuse of sensitive data [14, 15, 20, 24, 27], our findings highlight that questions about the clinical utility and value of CP technologies are equally important. Adolescents and caregivers seek clear justifications for the data collection enabled by CP tools, questioning whether the clinical benefits outweigh the privacy risks associated with generating large amounts of digital behavioral data. Both groups also wanted to know how inferences will be personalized to account for significant diversity and variability in the meaning (clinical significance) of behavioral patterns.

These knowledge needs echo ethical considerations highlighted by Shen et al. [29], who recently called for ethical frameworks to safeguard patient rights and address procedural and regulatory inconsistencies in CP research. They proposed a checklist of ethical considerations covering issues like those related to equity, diversity, access, privacy, research-industry partnerships, legal concerns, and return of results. Three of their key questions focused on establishing and adapting informed consent to meet the specific needs of various study populations, including surrogate decision makers. This paper provides the first empirical insights into the informational needs of patients and caregivers considering CP-related approaches into care. Although focused on adolescents in clinical research these findings are likely relevant across different study populations. In Table 10, we present an Informational Needs checklist aimed to guide providers and researchers in providing information to patients and research participants during patient education and informed consent, followed by suggestions and challenges for effectively addressing these needs. We hope that this checklist serves as a guideline, rather than an exhaustive or mandatory checklist, for providers to tailor their discussions based on a patient’s needs and values. Our hope is to support the delivery of patient-centered care while still remaining responsive to specific informational needs of patients and caregivers.

**Table 10:** Checklist of Stakeholder Informational Needs about Integrating Computer Perception Technologies into Clinical Research and Care.

Informational Domain	Patient Question Inventory
**Clinical Utility and Value** Questions about benefits, types of feedback to expect, and capacity to detect current and future disorder	a. What improvements do these tools provide compared to conventional care?
	b. What kind of specific, clinically relevant insights (feedback) can I expect (e.g., Summaries? Raw data? Recommendations? How often)?
	c. How are interpretations of the data tailored to me and my specific symptoms and illness experience?
	d. Can these tools detect disorders (present and future) that I don’t know about?
	e. How do my doctors translate information from these tools into decisions about my treatment?
**Evidence, explainability, evaluation and contestation** Questions about algorithmic explainability, types of evidence available, how physicians confirm algorithmic conclusions, right to redress/contestation	a. Will/can my physician explain to me how these algorithms work and arrive to conclusions?
	b. Have these capabilities been proven? What forms of proof/evidence (e.g. raw data; confidence statistics) should I be on the lookout for?
	c. What information/signals are these tools attending to or picking up about me / my patient, and how do they know what they mean for me and my condition?
	d. What other sources of information will my doctors gather to confirm that the information and inferences from these tools are correct? Where do they look to know they might be wrong?
	e. What can I do to contest or seek redress if I do not agree with clinical inferences made from my data?
**Accuracy and Trustworthiness** Questions about the trustworthiness of CP tools, generalizability across conditions, algorithmic bias, accounting for context and subjectivity, methods for evaluating trustworthiness	a. What can my doctor tell about my health/condition from these data?
	b. How much does my doctor rely on these data to inform my treatment? Does it replace his/her usual methods?
	c. How accurate is the information or conclusions these tools convey about me?
	d. How do I know my feedback/results aren’t biased (on the basis of gender, race, ethnicity, etc.?)
	e. Were people like me involved in developing these tools? (or are they similar/different from me in ways that I might want to know about)?
	f. How do my doctors take into account context and my own interpretations when making sense of my data?
**Data security, privacy, misuse** Concerns about the duration and type of data collection, data access and portability, data sharing and protection, linkage with other personal data, legal ramifications	a. For how long will data be collected (i.e., how much data)?
	b. What data will be collected (which behaviors), tracked or monitored?
	c. Who else (e.g., parents/caregivers; physician; clinical team; emergency services) will be able to access my data (and know when I am not doing well and need help?) (see also security)
	d. What will be done with these data (for clinical objectives; clinical research objectives; other?)
	e. How will these data be protected (using what mechanisms)?
	f. What links will be drawn for my data from these wearable/implantables to other sources of data about me (e.g., my social media usage, GPS)? If so, what concerns should I be aware of?
	g. How might these data be used against me in unwanted/unintended ways? (for example, by future employers, in education, or in the justice system?)h. What is the range of information being tracked?
	h. Who decides whether/which of my data is shared with my healthcare provider and/or others beyond my clinical team?
	i. How can I confirm where and how my data is being shared?
**Patient Consent, Control and Autonomy** Questions about transparency around data collection, patient and guardian rights, consent, control over data collection and feedback	a. How will I know that these tools are being used in my care, and for what purposes (e.g. diagnosis, symptom monitoring, risk prediction, etc.)? (Is my physician obligated to tell me?)
	b. As a child/adolescent, do I have a say over whether I want to use these tools in my healthcare or refuse/discontinue data collection?
	c. How can I be sure that my device(s) is (are) no longer collecting data if I don’t want it to?
	d. To what extent will I be able to access and/or possess my own data, including raw data, summaries, or both (or neither)?
	e. Do I (and/or my caregivers, clinicians, etc.) get to decide what feedback I want, when and/or how often?
	f. Will I (or my caregiver or doctor) be alerted if anything abnormal is picked up?
**Physician-Patient Roles and Relationship** *Concerns around preserving patient-centered care, impacts on the patient-doctor relationship, strategies for conflict resolution*	a. What steps will my physician take to make sure that I – and not just my data – remain at the center of my care?
	b. How might the use of CP impact my relationship with my physician (e.g., human connection and empathy)?
	c. How will my physician handle a situation when data patterns contradict what I say are my own subjective thoughts, feelings and behaviors?
**Patient Safety, Security, Well-being, and Dignity** Questions about regulatory oversight, customization of patient monitoring, and stigma/dignity concerns, possible negative impacts	a. Which regulatory entities (e.g. FDA) have approved these technologies for use?
	b. How will monitoring of my well being and security be tailored to how I specifically express distress or discomfort?
	c. Will my friends/family/colleagues be able to tell that I am wearing a device for CP? (stigma concerns)
	d. What negative consequences may arise if these tools are wrong about me and my condition?

### Clearly convey clinical utility of CP

The emphasis on the clinical utility and value of CP in care reflects a desire to better understand the risk-benefit calculus of using sensitive and private information beyond traditional clinical settings. Caregivers, all biological parents in this study, seek clear clinical justifications for using an intervention they view as new and investigational. This finding is noteworthy given the strong emphasis in the literature on privacy as a central ethical concern of these technologies and a relative under-emphasis on demonstrating clinical utility of CP tools for benefiting patient care [16, 32, 33]. While patients using CP may exchange privacy for effective treatment as is done in other areas of medicine (e.g., gynecology [34]; gastroenterology [35]), they are asked to trust CP’s invasiveness without sufficient empirical evidence of its benefits. As CP technologies move from research to regular care, patients are likely to demand stronger clinical justification for data collection outside the clinic. Providers must demonstrate that CP offers significant informational advantages over conventional methods and clarify for which conditions CP is appropriate.

### Address trustworthiness concerns

Both respondent groups also had questions about the validity of CP inferences, i.e., whether they accurately reflect a patient’s subjective experiences and behaviors. Many sought to understand how algorithms generate conclusions (explainability), how interpretation of outputs is complicated by patient heterogeneity and comorbidities, and how these outputs affect providers’ interpretations of a patient’s condition or risk for future conditions (interpretability and actionability). They also had questions about how providers and patients could evaluate or contest the accuracy and validity of CP outputs (contestability). Adolescents were more concerned than caregivers about understanding how conclusions are made and wanted the ability to challenge outputs that may contradict subjective experience. Both groups also questioned how CP technologies might impact the patient-provider relationship, especially if providers prioritize algorithmic insights over patient perspectives, particularly when these conflict. For some, this raised concerns about the dehumanization of care through an overreliance on technology; others seemed to want to retain epistemic authority over the meaning of their symptoms and experiences.

These findings reflect a justified reluctance to trust algorithmic interpretations over patients’ own understanding of their condition. Current limitations in algorithmic accuracy, such as bias in training data, lack of generalizability; data gaps, and misuse of proxies, necessitate careful interpretation of CP results [36–39]. Researchers and providers must be prepared to clearly communicate the accuracy, relevance and epistemological limitations of these tools, including their potential for bias, ensuring that patients understand these constraints. Since CP outputs remain investigational, many lack demonstrated validity, reliability and generalizability beyond small datasets. Further the assumption that these are objective metrics remain contested. Their clinical utility remains unverified and likely varies by condition and patient groups, which limits providers’ ability to reassure patients of the accuracy and validity of CP outputs.

Further, even if outputs were indeed valid, accurate and generalizable, research is lacking on how to effectively communicate their meaning in patient education and care. Outputs from CP algorithms, like those from other statistical and machine learning models, typically consist of (e.g., risk) probabilities calculated from numerous, quantified behavioral observations. Research on risk communication suggests that when confronted with probabilistic risk estimates, patients tend to overestimate [40] or underestimate their risk [41], and struggle to integrate multiple probabilities into an overall understanding of risk [40]. These difficulties may be influenced by health literacy [20] or the presence of a clinician who can help interpret algorithmic outputs, similar to a genetic counselor. Emerging literature [42, 43] suggests that algorithmic outputs should include additional information, such as risk variation across patient populations or clinical sites; confidence intervals; explanatory variables, and alternative information sources, to enhance and contextualize interpretations. Multistakeholder collaboration across the development, evaluation, and deployment of CP tools, might increase contract validity and mitigate sources of bias, which can inform best practices of communicating results and providing feedback to patients. More research is needed to determine how best to present these outputs in ways that facilitate understanding for both patients and providers, enabling clinical action where appropriate.

Providers should also be ready to address conflicts between algorithmic outputs and patients’ experiences. Several respondents expressed a desire to challenge algorithmic conclusions that could impact diagnosis and treatment plans; however, it remains unclear how best to reconcile conflicts between algorithms and patients, as well as between algorithms and providers. No protocols yet exist for handling scenarios where an algorithmic output aligns with a provider’s human judgment but not a patient’s, or when outputs from a validated algorithm challenge a provider’s judgment but perhaps align with a patient’s experience. Further research and ethical consideration are required to equip clinical and research teams to manage these conflicts and explore whether patients’ rights to challenge validated algorithmic conclusions differ from their rights to contest traditional clinical assessments.

### Provide patients with a roadmap for integrating CP into clinical care/research

Adolescents in particular raised questions about the role of CP data in clinical assessments and decision-making, and wondered how much access they would have to the clinical inferences from their data. Some feared that too much or too frequent might be disruptive, while too little might feel like surveillance without benefit. These concerns highlight varying preferences for feedback, suggesting that clinical teams should tailor feedback strategies based on individual needs. As we have argued elsewhere [22], personalized “roadmaps” balancing patient preferences with clinical reasoning may enhance integration of CP into care.

### Advocate for and participate in maintaining data privacy and protections

Our findings also emphasize the need to assure patients that their data will be securely collected, stored, and protected from unwanted disclosure. Both groups expressed concerns about data security, privacy, and misuse. Providers and researchers should inform patients about data use and the risks of unintended disclosure [15, 25]. However, their ability to do so is limited by providers’ own awareness of and ability to mitigate such risks, and varying institutional data security norms and practices, and current guidelines for managing CP data remain unclear, spurring some researchers (e.g., Muurling et al. [20]) to call for greater attention to a harmonization of research methodologies and data management and responsible stewardship of CP data. The characteristics of these data are often different from more traditional forms of health data in that they are not always directly or exclusively clinical in nature and often do not receive the same legal protections, leaving them more vulnerable to misuse by third-party controllers (e.g., targeted marketing, discrimination or exploitation) [44]. Further, they are often generated in the content of research/industry partnerships, leading to uncertainties about data storage, ownership rights and decision-making rights, and gaps in legal protection under the Health Insurance Portability & Accountability Act (HIPAA [45]) in the U.S. or Europe’s General Data Protection Regulation (GDPR [46]). This could be problematic for the several patients and caregivers who wanted to know what resources for legal redress might exist in cases of unintended use or disclosure of sensitive data or inferences, as to date there seem to be few.

Similarly, the growing risk of re-identification, where de-identified data can be traced back to individuals, further challenges data security efforts [47, 48]. Providers and researchers often lack the cybersecurity expertise needed to address these vulnerabilities effectively, suggesting a need to consult with specialized bioinformatics and cybersecurity professionals in clinical research or care involving CP. Research teams should allocate resources to include these experts, while providers should advocate for institutional support to mitigate data risks. Addressing these challenges will likely require significant shifts in funding and prioritization to protect patient data and ensure ethical data stewardship.

### Limitations

While our study provides a broad range of informational needs expressed by patients and caregivers regarding the ethical integration of CP tools in healthcare, the findings may not be generalizable to patients with diverse clinical and demographic profiles. We interviewed adolescents with a range of psychiatric diagnoses, along with their adult caregivers, across two different urban clinical sites. Further research is needed to explore informational needs that may be unique to individuals across other geographical regions, age groups, and condition types.

## Conclusion

This study provides an empirically derived checklist of key informational needs expressed by adolescents and caregivers regarding the use of CP tools in clinical research and care. This checklist is intended to help providers and researchers anticipate and address questions critical for ensuring informed consent. However, uncertainties around the clinical utility of CP tools, knowledge gaps in how best to communicate these issues to patients, and evolving data control and cybersecurity challenges limit providers’ and researchers’ ability to fully address many of these concerns. This study offers suggestions to help address these questions within the current landscape of clinical knowledge, risk communication approaches and data protections relevant to CP technologies.

### Citation diversity statement

The authors have attested that they made efforts to be mindful of diversity in selecting the citations used in this article.
